# Genome assembly and gene annotation of *Babesia ovis* Israeli strain (Piroplasmida: Babesiidae) using PacBio HiFi sequencing

**DOI:** 10.1038/s41597-026-08397-4

**Published:** 2026-09-30

**Authors:** Guilherme Borges Dias, Gisele Albuquerque, Hannes Yngve, Monica L. Mazuz, Ana Domingos, Sandra Antunes

**Affiliations:** 1 Department of Cell and Molecular Biology, National Bioinformatics Infrastructure Sweden, Science for Life Laboratory, Uppsala University, Uppsala, 752 37 Sweden; 2 Instituto de Higiene e Medicina Tropical – Universidade NOVA de Lisboa, UNL, Rua da Junqueira 100, 1349-008 Lisboa, Portugal; 3 Uppsala Genome Center, Science for Life Laboratory, Dept. of Immunology, Genetics and Pathology, Uppsala University, BMC, Box 815, SE-752 37 Uppsala, Sweden; 4 Kimron Veterinary Institute, P.O. Box 12, Bet Dagan, 50250 Israel; 5 Global Health and Tropical Medicine, GHTM, Associate Laboratory in Translation and Innovation Towards Global Health, LA-REAL, Instituto de Higiene e Medicina Tropical, IHMT, Universidade NOVA de Lisboa, UNL, Rua da Junqueira 100, 1349-008 Lisboa, Portugal

## Abstract

*Babesia ovis* is a unicellular parasite of sheep and goats transmitted by the tick *Rhipicephalus bursa*. *B. ovis* infection is widespread and can be lethal, and therefore there is need for a deeper understanding of its biology and pathogenesis. There is currently a single genome available for a clinical isolate of *B. ovis*, and no laboratory strains have been sequenced so far. In this study we have sequenced and assembled the genome of the Israeli laboratory strain of *B. ovis* using highly accurate long-read sequencing (PacBio HiFi). The resulting assembly has high contiguity, with the four largest contigs comprising 96% of the assembly and representing putative near-complete chromosomes. Annotation identified 3,549 genes, of which 3,046 (85.6%) could be functionally annotated. BUSCO analysis on the annotated genes revealed 95.5%, 98.8% and 100% completeness of the Aconoidasida, Apicomplexa, and Alveolata lineages, respectively. Comparative analysis with other annotated piroplasmida genomes reveals the vast majority of predicted genes clustered in orthogroups. The availability of a high-quality genome assembly for a tractable laboratory strain could prove impactful in unlocking more basic and applied research avenues to be pursued for this important parasite system.

## Background & Summary

*Babesia ovis* is a tick-borne apicomplexan parasite that infects sheep and goats, primarily in Mediterranean, Middle Eastern, and parts of Central Asian regions^1,2^. Transmitted by the tick *Rhipicephalus bursa*, *B. ovis* is responsible for ovine babesiosis, a disease that can cause severe clinical outcomes such as fever, anemia, hemoglobinuria, and high mortality rates, especially in naïve or young animals^2^. The disease poses significant economic burdens due to loss of livestock, reduced productivity, and treatment costs. Despite its impact on animal health, *B. ovis* remains poorly understood at the molecular level. One of the major limitations in advancing *B. ovis* research is the scarcity of high-quality genomic resources. Genomic data are essential for understanding parasite biology, pathogenesis, antigenic diversity, and drug resistance mechanisms, as well as for the development of effective diagnostics and vaccines^3^. To date, only one genome sequence of *B. ovis*—from a field-derived clinical isolate—has been made publicly available^4^. However, the absence of genome assemblies from laboratory-adapted strains has constrained both functional genomics and comparative analyses. Laboratory strains are particularly valuable for experimental validation, molecular manipulation, and standardized biological assays, all of which require well-characterized, stable reference genomes^5^. To help close this gap, our study aimed to generate a high-quality, chromosome-level genome assembly of the Israeli laboratory strain of *B. ovis*. We employed state-of-the-art long-read sequencing technology (PacBio HiFi), which is particularly well-suited for resolving repetitive and AT-rich regions commonly found in apicomplexan genomes. The approach combined *in vitro* culture of *B. ovis*, high-purity DNA extraction, and state-of-the-art assembly and annotation pipelines. By producing a comprehensive genome resource from a lab strain, our goal was to provide a standardized and genetically tractable reference for future molecular and evolutionary studies. The resulting dataset will serve as a foundational genomic resource to support diverse areas of research, including population genomics, vaccine and drug target discovery, and host–pathogen interaction studies. It also opens the door to comparative genomic analyses within the *Babesia* genus and broader Apicomplexa phylum. Importantly, the availability of a well-annotated, experimentally accessible genome will facilitate integration of *B. ovis* into modern functional genomics workflows and could contribute to improved control strategies for ovine babesiosis.

## Methods

### Parasite culture and DNA extraction

*Babesia ovis in vitro* cultures were established in ovine erythrocytes under biosafety level 2 conditions as described elsewhere^6–8^. Briefly, a cryopreserved Israeli field isolate of *B. ovis*, originally obtained from a naturally infected sheep in Israel and maintained at the Kimron Veterinary Institute, Israel, was thawed and used to initiate the culture in 10% (vol/vol) defibrinated lamb red blood cells (RBCs). Parasites at passage 39 were used in the present study. The isolate was not cloned during its passage history. The base medium was HEPES-buffered Medium 199 (with Earle’s salts, L-glutamine, and amino acids) supplemented with 20% (vol/vol) ovine serum, antibiotic–antimycotic solution (final concentrations: 0.05 µg/mL amphotericin B, 20 U/mL penicillin, 20 µg/mL streptomycin), 1 mM L-cysteine-HCl, 2 mM bathocuproine-disulfonic acid, and 25 mM TES buffer (N-[tris(hydroxymethyl)methyl]-2-aminoethanesulfonic acid). Cultures were maintained at 37 °C under a microaerophilic atmosphere (5% CO_2_, 2% O_2_, 93% N_2_) in a modular incubator chamber. Culture medium was replaced daily, and parasitaemia was monitored by preparing thin blood smears stained with Hemacolor® rapid stain. Intraerythrocytic parasites were readily observed on daily smears under a 100 × oil-immersion objective (≈1000 × total magnification), confirming successful *in vitro* propagation of *B. ovis*. For DNA extraction, cultures with parasitaemia levels above 8%, typically reached 8–9 days after culture initiation, were used to maximize parasite DNA yield while limiting host DNA contamination, given the anucleate nature of ovine erythrocytes. The cultures were centrifuged to pellet the cells, and genomic DNA was extracted from the parasites using the DNeasy Blood & Tissue Kit (Qiagen, Hilden, Germany) according to the manufacturer’s instructions. For each extraction, a culture volume corresponding to approximately 10 µL of packed RBCs was used, estimated to contain ~3 × 10^^8^ erythrocytes (corresponding to at least ~2.4 × 10^^7^ parasitized erythrocytes at > 8% parasitaemia). The extracted DNA was eluted in 100 µL of buffer, and DNA concentration was measured using a Qubit 4 Fluorometer (Thermo Fisher Scientific, Waltham, MA, USA). DNA purity was assessed by UV spectrophotometry, recording the A_260_/A_280_ and A_260_/A_230_ absorbance ratios. Two independent *B. ovis* DNA samples were obtained from separate cultures (labeled DNA *B. ovis* 1 and DNA *B. ovis* 2). DNA *B. ovis* 1 had a concentration of 17.4 ng/µL, with A_260_/A_280_ = 1.91 and A_260_/A_230_ = 1.8 (in 100 µL volume). DNA *B. ovis* 2 yielded 14.2 ng/µL, with A_260_/A_280_ = 1.95 and A_260_/A_230_ = 1.8 (100 µL volume). These yield and purity values indicate that moderate amounts of parasite DNA were obtained, and the purity ratios (~1.9 for A_260_/A_280_ and ~1.8 for A_260_/A_230_) were within acceptable ranges, reflecting highly pure DNA suitable for genomic analyses.

### Library preparation and sequencing

Input QC of the DNA was performed using the Dropsense96 instrument (Trinean/Lunatic), Qubit 2.0 Fluorometer (Invitrogen) and FEMTO Pulse system (Agilent) to evaluate concentration, purity and size. Sample shearing was achieved using the Megaruptor 3 DNA Shearing System (Diagenode) at speed 30. An AMPure bead purification was performed after the shearing. The sample libraries were prepared according to the “Preparing whole genome and metagenome libraries using SMRTbell prep kit 3.0” (102-166-600 REV02 MAR2023) (Pacbio) using the SMRTbell prep kit 3.0 (PacBio 102-182-700). The samples were size selected using SMRTbell cleanup beads according to Pacbio’s protocol. Quality control of sheared DNA and SMRTbell libraries was performed on the Fragment Analyzer System (Agilent). Primer annealing and polymerase binding was performed using the Sequel II binding kit 3.2. The library was sequenced on the Sequel IIe instrument, using the Sequel II sequencing plate 2.0 and the Sequel II SMRT Cell 8 M with movie time 30 hours and pre-extension time 2 hours.

Sequencing generated 2,920,915 HiFi reads with a total yield of 28.8 Gbp. With an estimated genome size of less than 10 Mbp, this puts the HiFi theoretical coverage at approximately 2800x. Because genome assembly with extremely high coverage can be detrimental to quality, downsampling was performed. A random sample of reads representing 10% (approximately 280x genome coverage) of the original dataset was produced using seqtk (seed = 2) v1.3-r106 (available at https://github.com/lh3/seqtk).

### Genome properties and genome assembly

**Fig. 1:**
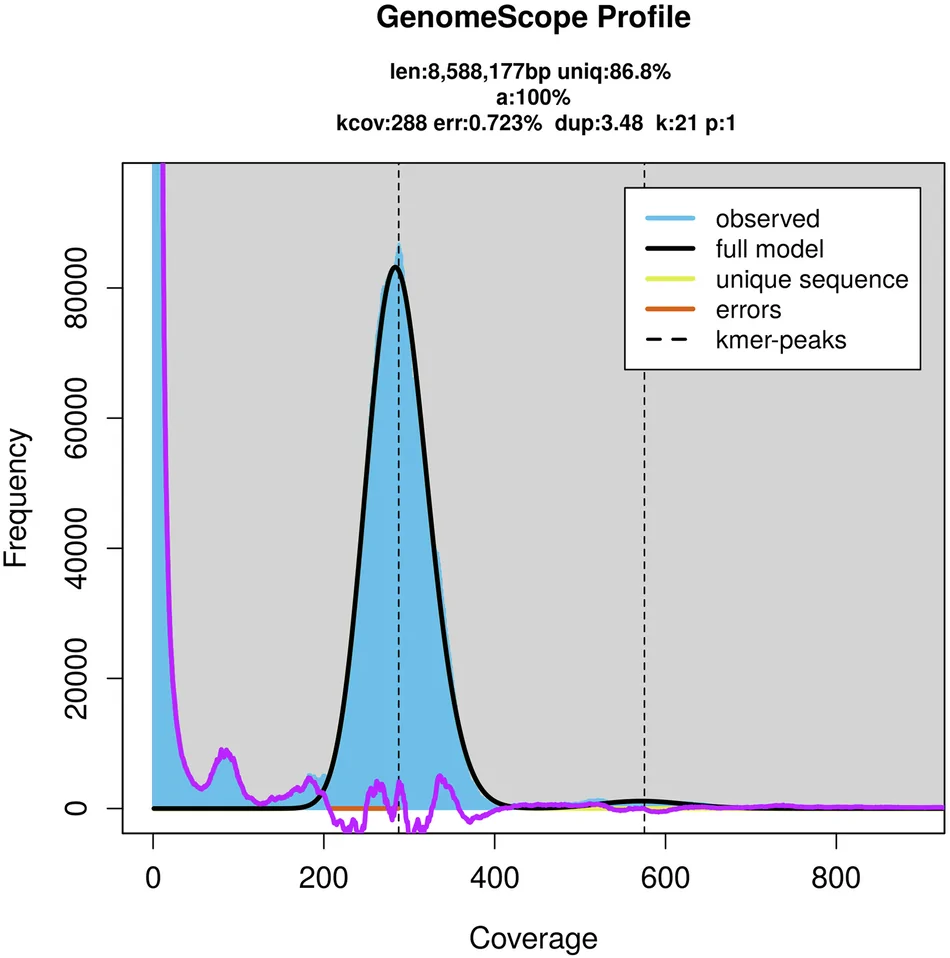
Genome size estimation for *Babesia ovis* (Israeli strain) from a subsample of the PacBio HiFi data. The main peak indicates coverage of 288x, assuming a haploid genome. A smaller peak can be observed at ~80x potentially indicating residual errors or host contamination.

GeneScopeFK^9^ was used to estimate genome size, using as input the histogram of 21-mer counts produced from the subsampled HiFi reads with FastK v1.2 (available at https://github.com/thegenemyers/FASTK). This analysis revealed a peak at 288x coverage representing the haploid genome, and an additional peak at 80x possibly representing residual sequencing errors or host DNA contamination (Fig. 1). The k-mer-based estimated genome size was 8.59 Mbp, with 87% unique and 13% repetitive portions. The fraction of repeats roughly matches the results of the RepeatMasker step performed for annotation (see below).

Hifiasm v0.19.8^10^ was used to assemble the downsampled HiFi reads with default parameters. The resulting draft assembly was scanned for contamination using the blobtoolkit pipeline v4.1.7^11^, and contigs with a taxonomic assignment other than Apicomplexa were removed. In addition, two short contigs matching Apicomplexa but with lower coverage than expected (<100x) and devoid of any BUSCO genes (Alveolata, Apicomplexa, and Aconoidasida OrthoDB v10) were also removed.

Since no mitochondrial contig was detected in the de novo assembly, we used the related *B. bovis* mtDNA (NC_009902.1) as a reference to map PacBio HiFi reads with minimap2 v2.24-r1122^12^. We then extracted mapped reads with samtools v1.7^13^, and assembled them using MBG v1.0.16^14^. The resulting assembly is a linear sequence of 6,067 bp.

One contig, ptg000012c (109,501 bp), had a comparatively low GC content (20.6%) and was flagged as being circular by hifiasm. This contig included approximately 3 tandem copies of the apicoplast genome. In order to recover a single complete apicoplast genome we first mapped all reads to contig ptg000012c using minimap2 v2.24-r1122^12^ and then assembled the mapped reads using MBG v1.0.16^14^. This resulted in a single circular sequence of 34,948 bp (JBOFAQ010000010.1).

The final decontaminated assembly had a length of 9 Mbp and comprised 9 contigs plus the apicoplast and mitochondrial segments. Importantly, the four longest contigs span 8.67 Mbp or 96% of the total assembly, matching the expected number of chromosomes for *B. ovis* in its haploid genome (n = 4).

### Genome annotation

Prior to structural annotation we created a species-specific repeat library using the RepeatModeler2 v2.0.2a package^15^. From this repeat library, identification and masking of repeat sequences present in the genome was performed using RepeatMasker v4.1.5 (available at https://www.repeatmasker.org/). With this approach, approximately 16% of the genome was masked, roughly matching the predicted repetitive fraction from k-mer frequency analysis (Fig. 1).

Structural annotation was performed on the repeat-masked genome with Braker v3.0.3^16^ incorporating external evidence in the form of all alveolata proteins from OrthoDB v11^17^ (https://bioinf.uni-greifswald.de/bioinf/partitioned_odb11). Redundant gene models with source “GeneMark.hmm3” were removed.

The predicted genes were functionally annotated using the NBIS functional_annotation nextflow pipeline v2.0.0 (https://github.com/NBISweden/pipelines-nextflow). Briefly, this pipeline performs similarity searches between the annotated proteins and the UniProtKB/Swiss-Prot database (downloaded on 2022-12; 568,363 proteins) using BLAST. Then it uses InterProScan^18^ to query the proteins against InterPro v59-91 databases^19^, and merges results using AGAT v1.2.0^20^.

Functional annotation of the predicted genes with Interproscan detected a large number of genes (3,046 out of 3,549) that could be associated with functional information from one of the InterPro databases.

Transfer RNAs (tRNAs) were annotated using tRNAscan-SE v2.0.12^21^. This analysis identified 46 tRNA genes. Ribosomal RNAs (rRNAs) were annotated using barrnap v0.0.9 (available at https://github.com/tseemann/barrnap). This analysis identified 13 rRNA genes including 3 copies of 18S, 5.8S, and 28S rRNAs, and 4 copies of 5S.

### Comparative analysis

To gain further insight into the quality of gene annotation and the utility of this genome for comparative studies we have analysed the predicted proteome for *Babesia ovis* in the context of other available annotated piroplasmida proteomes in GenBank and RefSeq. The selected species were *Babesia ovis* isolate Selcuk (GCA_023705535.2), *Babesia microti* strain RI (GCA_000691945.2), *Babesia duncani* isolate WA1 (GCA_028658345.1), *Babesia caballi* isolate USDA-D6B2 (GCA_024862765.1), *Babesia ovata* strain Miyake (GCA_002897235.1), *Babesia bigemina* strain Bond (GCA_000981445.1), *Babesia bovis* strain T2bo (GCA_000165395.2), *Babesia* sp. strain Xinjiang (GCA_002095265.1), *Theileria equi* strain WA (GCA_000342415.1), *Theileria orientalis* strain Shintoku (GCA_000740895.1), *Theileria annulata* strain Ankara isolate clone C9 (GCA_000003225.1), *Theileria parva* strain Muguga (GCA_000165365.1), and *Plasmodium falciparum* isolate 3D7 (GCA_000002765.3) as an outgroup. The comparative analysis was performed using OrthoFinder v2.5.5^22^ with default parameters.

**Fig. 2:**
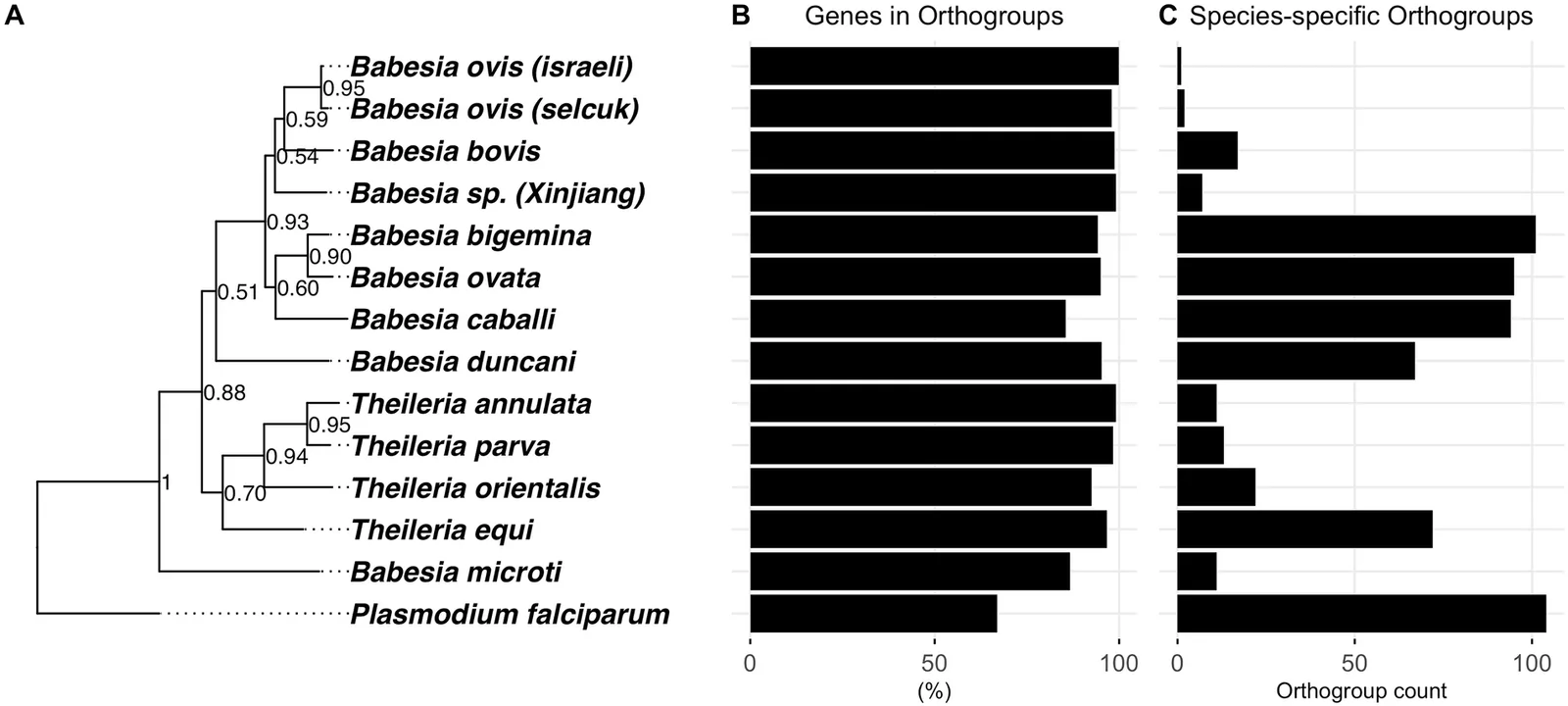
Orthogroup analysis of *Babesia ovis* in the context of other piroplasmida genomes. (**A**) Species tree inferred from orthogroup gene trees with STAG^22^ and rooted using inferred gene duplications with STRIDE^33^. *Plasmodium falciparum* was included as an outgroup to piroplasmida. (**B**) Percentage of genes clustered in orthogroups for each species. (**C**) Number of species-specific orthogroups.

The analysis of shared orthologous groups correctly recovers the inferred piroplasmida phylogeny, including the paraphyly of *B. microti*^23^ (Fig. 2A). *B. ovis* (Israeli) forms a clade in the tree with *B. ovis* (Selcuk), and *B. bovis* is the next closest species. Nearly all *B. ovis* predicted genes cluster in orthogroups with other piroplasmida genomes (Fig. 2B) and very few species-specific orthogroups are identified, likely because of the inclusion of the Selcuk isolate (Fig. 2C).

## Data Records

Data generated in this study, including the raw PacBio HiFi reads and genome assembly, were deposited to the National Center for Biotechnology Information (NCBI). The raw PacBio HiFi reads are available in the Sequence Read Archive (SRA) under accession SRP521891^24^. The whole-genome assembly is available in GenBank under accession GCA_051529585.1^25^. The predicted gene models are available in Figshare^26^.

## Technical Validation

**Fig. 3:**
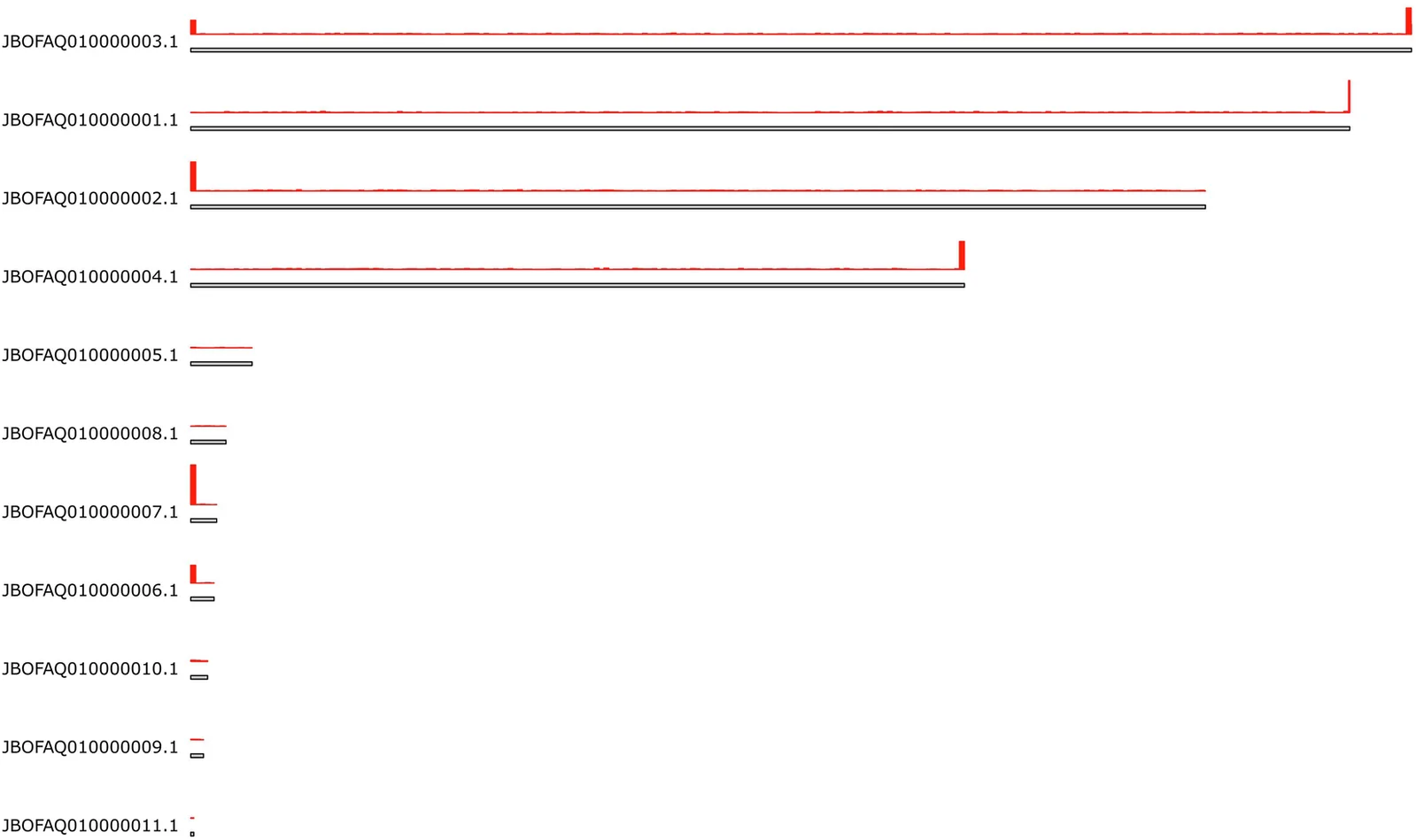
Telomere analysis of *Babesia ovis* (Israeli strain) genome assembly. The abundance of the apicomplexa telomere motif “CCTAAA”^28^ was measured in non-overlapping 10 kb windows and plotted as red bars along the assembled contigs.

The final assembly has a contig N50 of 2.41 Mbp. We inspected the assembled contigs for the presence of telomeric sequences using tidk v0.2.31^27^ and the Apicomplexa telomeric motif “CCTAAA”^28^. The presence of the motif was visualised with karyoploteR v1.26.0^29^. This analysis highlights the presence of the telomeric motif on both ends of JBOFAQ010000003.1, indicating this might be a complete chromosome. The telomere is also observed on the right end of JBOFAQ010000001.1 and JBOFAQ010000004.1, and on the left end of JBOFAQ010000002.1. In addition, two short contigs, JBOFAQ010000006.1 and JBOFAQ010000007.1 also present the telomeric motif on their left end (Fig. 3).

**Fig. 4:**
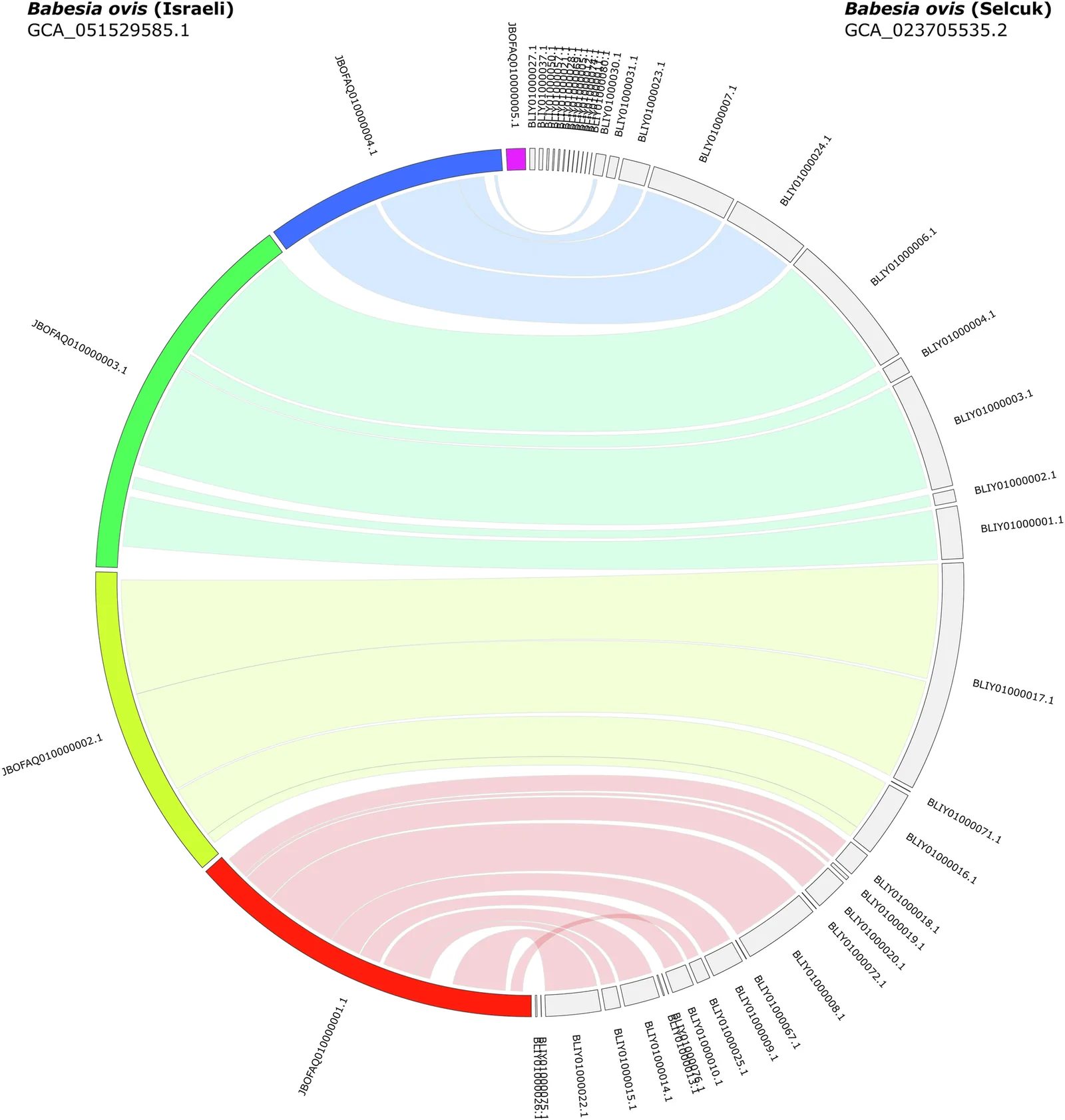
Circos consistency plot between the assemblies of two strains of *Babesia ovis* (Israeli and Selcuk). Colored rectangles on the left-hand side of the image represent the contigs from the Israeli strain assembly whereas grey rectangles on the right-hand side represent scaffolds from the Selcuk strain.

To investigate potential misassemblies we have aligned the Israeli strain assembly to another assembly generated for an isolate of *B. ovis* from Turkey (GCA_023705535.2, isolate Selcuk) generated using Nanopore, PacBio, and Illumina data^4^. This comparison was performed with a Circos consistency plot^30^ and indicates no large-scale discordances between both assemblies, with the Israeli strain possessing greater contiguity compared to Selcuk (Fig. 4).

Completeness of the assembly and annotation (predicted proteins) was assessed using BUSCO v5.4.6^31^ with default parameters. Three BUSCO lineages from OrthoDB v10^32^ were used, the Alveolata (n = 171), Apicomplexa (n = 446), and Aconoidasida (n = 1,135). Completeness is high and duplication low for both the assembly and annotation (Table 1). Interestingly, the completeness is higher in the annotation than in the assembly itself, especially for the aconoidasida gene set (Table 1). This is also observed for many other piroplasmida genomes (BUSCO stats available at https://piroplasmadb.org/), and could reflect sensitivity limitations of BUSCO on genome mode compared with evidence-based gene annotation.

**Table 1:** Benchmarking Universal Single-copy Orthologs (BUSCO) analysis of *Babesia ovis* (Israeli) genome and annotation.

BUSCO statistic	Lineage		
	alveolata (n = 171)	apicomplexa (n = 446)	aconoidasida (n = 1,135)
Complete	99.4 / 100	96.4 / 98.8	83.6 / 95.5
Single-copy	99.4 / 99.4	96 / 97.5	83.2 / 93.4
Duplicated	0 / 0.6	0.4 / 1.3	0.4 / 2.1
Fragmented	0.6 / 0	0.7 / 0.4	5.2 / 1.2
Missing	0 / 0	2.9 / 0.8	11.2 / 3.3

BUSCO statistics (%) are presented for each lineage gene set with the first value before the slash referring to the genome and the second value referring to the annotation.

## Data Availability

All software used in this project was executed with default options for their respective version unless otherwise noted in the methods. Command line arguments used are available in GitHub (https://github.com/gbdias/b_ovis_supplementary_code).
